# Population Toxicokinetic Modeling of Cadmium for Health Risk Assessment

**DOI:** 10.1289/ehp.0800317

**Published:** 2009-05-06

**Authors:** Billy Amzal, Bettina Julin, Marie Vahter, Alicja Wolk, Gunnar Johanson, Agneta Åkesson

**Affiliations:** 1 Assessment Methodology Unit, European Food Safety Authority, Parma, Italy; 2 Unit of Nutritional Epidemiology; 3 Unit of Metals and Health and; 4 Unit of Work Environment Toxicology, Institute of Environmental Medicine, Karolinska Institutet, Stockholm, Sweden

**Keywords:** alternative model development, Bayesian inference, cadmium toxicokinetics, population variability, risk assessment, toxicokinetic models, urinary cadmium

## Abstract

**Background:**

Cadmium is a widespread environmental pollutant that has been shown to exert toxic effects on kidney and bones in humans after long-term exposure. Urinary cadmium concentration is considered a good biomarker of accumulated cadmium in kidney, and diet is the main source of cadmium among nonsmokers.

**Objective:**

Modeling the link between urinary cadmium and dietary cadmium intake is a key step in the risk assessment of long-term cadmium exposure. There is, however, little knowledge on how this link may vary, especially for susceptible population strata.

**Methods:**

We used a large population-based study (the Swedish Mammography Cohort), with repeated dietary intake data covering a period of 20 years, to compare estimated dietary cadmium intake with urinary cadmium concentrations on an individual basis. A modified version of the Nordberg-Kjellström model and a one-compartment model were evaluated in terms of their predictions of urinary cadmium. We integrated the models and quantified the between-person variability of cadmium half-life in the population. Finally, sensitivity analyses and Monte Carlo simulations were performed to illustrate how the latter model could serve as a robust tool supporting the risk assessment of cadmium in humans.

**Results:**

The one-compartment population model appeared to be an adequate modeling option to link cadmium intake to urinary cadmium and to describe the population variability. We estimated the cadmium half-life to be about 11.6 years, with about 25% population variability.

**Conclusions:**

Population toxicokinetic models can be robust and useful tools for risk assessment of chemicals, because they allow quantification and integration of population variability in toxicokinetics.

Cadmium is a widespread environmental pollutant (Nordberg et al. 2007). There is accumulating data of toxic effects on kidney (Buchet et al. 1990; de Burbure et al. 2003; Järup et al. 2000; Nishijo et al. 2006; Suwazono et al. 2006) and bone (Åkesson et al. 2006; Alfven et al. 2004; Gallagher et al. 2008; Schutte et al. 2008; Staessen et al. 1999) at urinary cadmium concentrations < 2.5 μg/g creatinine, previously considered to represent a safe exposure [Joint Food and Agriculture Organization of the United Nations (FAO)/World Health Organization (WHO) Expert Committee on Food Additives 2003]. A precautionary approach in the risk assessment of cadmium is further supported by recent findings suggesting increased risk of cancer and overall mortality in relation to low-level cadmium exposure (Åkesson et al. 2008; Nawrot et al. 2006, 2008), as well as variation in susceptibility (Vahter et al. 2007).

Diet is the major source of cadmium exposure in nonsmokers (Nordberg et al. 2007). The concentrations of cadmium in food vary considerably, but often foods of plant origin, such as cereals, potatoes, roots, and vegetables, are the major contributors to the exposure (Olsson et al. 2002). Cadmium accumulates mainly in the kidneys, and the kidney concentration of the metal is reflected in the concentration in urine, which thereby can be used as a marker of long-term cadmium exposure (Nordberg et al. 2007). Urinary cadmium concentrations are therefore commonly used as a surrogate for the body burden in health risk assessment. In order to perform a reliable and comprehensive assessment of the health risks associated with long-term exposure to cadmium in food, data on dietary intake of cadmium need to be related to the internal dose over many years.

To assess the variation in the association between dietary cadmium intake and urinary cadmium in the general population, various individual physiologic parameters affecting the internal dose of cadmium need to be taken into account. Parameters such as gastrointestinal absorption and biological half-life (Nordberg et al. 2007) affect the target dose of cadmium in the body and may vary among individuals. Toxicokinetic (TK) models have been developed for cadmium, such as the Nordberg-Kjellström’s physiologically based toxicokinetic (PBTK) model (Kjellström 1971; Kjellström and Nordberg 1978) and further developments thereof [see Supplemental Material (doi: 10.1289/ehp.0800317.S1)], hereafter designated as the “eight-compartment model.” A simpler one-compartment model has been used to predict urinary cadmium from food-cadmium intake [Kjellström 1971; WHO/International Program on Chemical Safety (IPCS) 1992]. Neither model has, however, been tested or validated in humans based on individually paired dietary and urinary cadmium data. Furthermore, the quantification of the between-person variability in a population is a crucial issue in risk assessment in order to build a robust statistical link between the urinary concentrations and the intake. Intervariability in a population has never been fully integrated or quantified in the models from the literature, leaving risk assessors with the use of uncertain safety factors in order to protect a given proportion of the population.

Based on new individual data of estimated long-term dietary cadmium intake and measured urinary cadmium concentrations, the aims of the present study were to *a*) quantitatively assess and compare the eight-compartment PBTK model with the one-compartment TK model in terms of individual urinary cadmium predictions; *b*) integrate in the model and quantify the between-person variability in the population of cadmium toxicokinetics; and *c*) estimate the population distribution of daily cadmium intakes corresponding to a pre-defined urinary cadmium concentration. The overall aim is to refine cadmium risk assessments in food and environment.

## Materials and Methods

### Study population

The Swedish Mammography Cohort (SMC) was established in 1987–1990, when all 90,303 women residing in two counties in central Sweden, and born between 1914 and 1948, received a mailed invitation to be screened by mammography. Enclosed with this invitation was a six-page questionnaire regarding diet, body size, and education; 74% of women completed the questionnaire. In the autumn of 1997, a second questionnaire, expanded to include about 350 items concerning diet and other lifestyle factors (including smoking habits), was sent to all 56,030 participants who were still alive and living in the study area; 39,277 women (70%) completed this questionnaire.

In 2004, a validation study of cadmium intake was initiated in a subcohort of women. After completion of a food frequency questionnaire (FFQ), women living in the town of Uppsala were successively invited for blood and urine sampling and their weight was recorded. Between 2004 and 2007, data from a total of 1,519 women (56–70 years of age) were collected. After excluding those with implausible values for total energy intake (three standard deviations from the mean value of log*_e_*-transformed energy intake; *n* = 26) and women who had ever smoked (*n* = 813), 680 women remained for the modeling analysis. The study was approved by the Regional Ethical Review Board in Stockholm, Sweden, and written informed consent was obtained from each participant.

### Dietary assessment

An FFQ with 67, 96, and 123 food items was used to assess diet at baseline in 1987–1990, in 1997, and in 2004–2007, respectively. The women were asked to report how often, on average, they had consumed each food item during the previous 6 months (baseline) or the past year (1997 and 2004–2007 FFQs). The questionnaires had eight mutually exclusive predefined categories for frequency of consumption, ranging from never/seldom to more than three times a day. For some commonly consumed foods such as bread, coffee, tea, and dairy products, there were open questions. The baseline FFQ has been validated in a sub-sample of 129 women randomly chosen from the study population. The Pearson correlation coefficients (*r*) between the FFQ and the mean of four 1-week weighted diet records were between 0.5 and 0.8 for the main cadmium-contributing foods. In a study of 57 Swedish women, dietary cadmium intake, assessed in duplicate diets, correlated well with tissue cadmium accumulation as assessed by the urinary excretion of the metal; *r* = 0.7 (Åkesson A, unpublished data).

Data of cadmium concentration in each of the different food items in the FFQ were obtained from the Swedish National Food Administration (NFA; Uppsala, Sweden) (Becker and Kumpulainen 1991; Jorhem and Sundstrom 1993; Jorhem et al. 2001), as previously described (Åkesson et al. 2008). In order not to compromise the quality of the data, we excluded analyses performed before 1980, with the exception of butter, margarine, and oils (analyzed 1976–1978), for which later results were lacking. For only a few food items (e.g., certain vegetables, fruits, and some types of bread) for which no Swedish data were available, Finnish (Tahvonen and Kumpulainen 1994, 1995) and Danish (Larsen et al. 2002) data were used. Cadmium content in prepared dishes was calculated based on a recipe database from the NFA and on the cadmium concentration in each ingredient. The exposures from air (< 1% of total exposure; Vahter et al. 1991) and drinking water (community-provided tap water and private wells; Olsson et al. 2002) were low and not included.

The individual average cadmium intake of the women in the study cohort was calculated based on the questionnaires used at baseline (1987–1990), 1997, and 2004–2007, by multiplying the frequency of consumption of each food item by its cadmium content per age-specific serving (≤ 65, > 65 years). The age-specific serving sizes were based on mean values from weighted foods recorded during four 1-week periods 3–4 months apart by randomly selected women from the cohort (Wolk A, unpublished data). We adjusted cadmium for total energy intake of 1,700 kcal using the residual-regression method (Willett 1998).

### Urinary cadmium assessment

The concentration of cadmium was determined in the first-void morning urine, which was collected in urine cups that had been tested free from contamination and transferred to nitric acid–washed 13-mL polypropylene tubes. To minimize the risk of cadmium contamination of urine, the women received detailed sampling instructions. The samples were stored upon arrival at the laboratory at −80°C until analysis. Cadmium concentrations were measured using inductively coupled plasma mass spectrometry (Agilent 7500ce, Agilent Technologies, Waldbronn, Germany) with the collision cell in helium mode. Cadmium solutions for external calibration were prepared fresh before every run from a 10.1 mg/L stock solution (ICP Multi Element Standard Solution VI CertiPUR; Merck, Darmstadt, Germany). The urine samples were thawed in room temperature and then diluted 10 times with 1% nitric acid. Cadmium isotope 111 was measured and calculations were made with ChemStation software (Agilent Technologies).

For quality control purposes, commercial reference materials (Seronorm Trace Elements Urine, ref. no. 201205, lot no. NO2525;Seronorm Trace Elements Urine Blank, ref. no. 201305, lot no. OK4636; SERO AS, Billingstad, Norway) were analyzed. Mean cadmium concentrations in the Seronorm samples were 4.80 ± 0.13 μg/L (*n* = 68; recommended value 5.06 ± 0.22) and 0.24 ± 0.03 μg/L (*n* = 121; recommended value 0.31 ± 0.05), respectively. The coefficients of variation (CVs) were 2.6% and 13.0%, respectively. The limit of detection, calculated as three times the standard deviation of the blank values, was 0.003 μg/L. Because TK models were available only for creatinine-adjusted data, all urinary cadmium concentrations were expressed as micrograms per gram of urinary creatinine.

### TK models

Two modeling approaches were investigated and compared. First, we considered a modified version of the eight-compartment PBTK model [Kjellström 1971; Kjellström and Nordberg 1978; see Supplemental Material (doi: 10.1289/ehp.0800317.S1)]. This model describes cadmium absorption, transport, and excretion via differential equations. Default gastrointestinal absorption fraction value was set to 0.1 (i.e., 10%). Red blood cell and plasma default volumes were set to 2.2 L and 3 L, respectively. Most of the parameter values were taken from Diamond et al.(2003), including the growth curve describing how body weight grows with age.

Second, we considered an alternative one-compartment model (Kjellström 1971). Such a simpler model focuses on the kidney accumulation and urinary excretion, making rough and global assumptions on the other pathways. In cases of poor prior knowledge of the physiologic parameters involved in cadmium kinetics, it allows a simplified and parsimonious description of cadmium excretion, hence facilitating further statistical evaluations, such as the evaluation of population variability or integration of intraindividual variability.

The one-compartment model considered here is a standard first-order elimination model with bolus administration (see, e.g., Gibaldi and Perrier 1982). For a given intake of cadmium (*d*_0_) at time 0, the accumulated amount of cadmium in the kidney at time *t* is calculated as


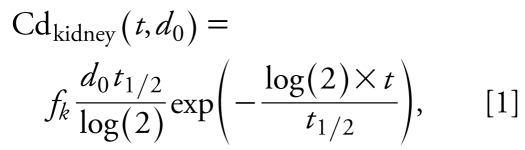


where *t*_1/2_ is the cadmium half-life and *f_k_* is a factor aggregating several physiologic and cadmium-related constants:


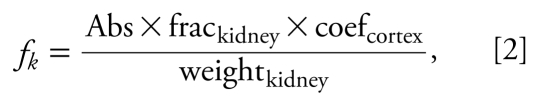


where Abs is the gastrointestinal absorption coefficient (%), frac_kidney_ is the fraction of cadmium transported to kidney (%), coef_cortex_ is the coefficient translating cadmium in the whole kidney into cadmium in the kidney cortex, and weight_kidney_ is the kidney weight (kilograms) assumed to be proportional to body weight.

We considered repeated exposure to cadmium as yearly bolus doses, from birth (year *i* = 0) until the current age (year *i* = *t*), as shown in Equation 3, where the sum is a yearly dietary addition of cadmium to the kidney accumulation, from birth until current age:


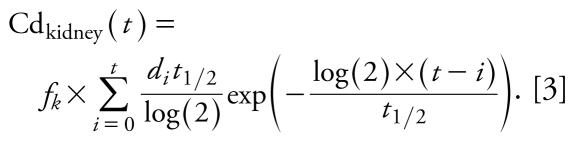


The urinary cadmium concentration was assumed to be proportional to the cadmium concentration in the kidney cortex, so the urinary cadmium concentration (in micrograms per gram of creatinine) at day *t* is obtained by


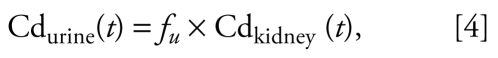


where *f_u_* is the ratio between cadmium in urine (micrograms per gram of creatinine) and in kidney cortex (micrograms per kilogram of kidney cortex).

Finally, in the case where dose per kilogram of body weight is assumed to be constant over the lifetime, the equation of the model can be simplified into the more classical one:


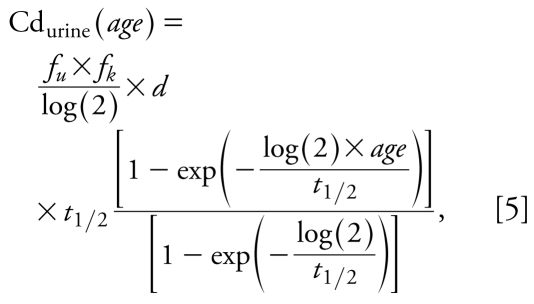


where *d* stands for the cadmium intake. This simpler form without any sum prevents numerical problems and can be derived after standard algebraic calculations under the assumption that cadmium intake per kilogram body weight did not vary over time.

Furthermore, we assumed that the daily cadmium intake was proportional to the body weight for each individual. This assumption is supported by the results from the third U.S. National Health and Nutrition Examination Survey (NHANES III) as reported by Choudhury et al. (2001), who observed no substantial difference among age groups in the daily cadmium intake per kilogram of body weight (except for young children). This assumption is further supported by the outcome of the trend analysis. We calculated for each individual the mean yearly cadmium intake over the three measurement periods as a proxy for the average long-term cadmium intake. The population mean of the cadmium half-life in the kidney was set to vary between 10 and 30 years. Such quantitative prior information as well as possible ranges for the parameter (*f_k_* × *f_u_*) were incorporated using Bayesian inference (described under “Statistical methods and models”).

We constructed the potential range for the aggregate factor *f_k_* coefficient based on the following biological data:

- We assumed the gastrointestinal absorption coefficient among women to range from 1% to 10% (Flanagan et al. 1978; Nordberg et al. 2007).
- The fraction of cadmium transported to kidney is generally estimated to be around one-third (Kjellström and Nordberg 1978; Nordberg et al. 1985).
- We assumed the coefficient translating cadmium in the whole kidney into cadmium in the kidney cortex to be 1.25 (Nordberg et al. 1985; Svartengren et al. 1986).
- We assumed the kidney weight to be 0.43% of total body weight, based on kidney weights of 0.235 kg and 0.3 kg at body weights of 55 kg and 70 kg, respectively (Kjellström and Nordberg 1978; Nordberg et al. 1985).

Furthermore, we based the potential range for the constant *f_u_* on the assumption that a cadmium concentration of 50 mg/kg in kidney cortex corresponds to 1.7–2.5 μg cadmium per gram of creatinine in urine (Kjellström and Nordberg 1978; Nordberg et al. 1985; Orlowski et al. 1998). The time step we chose for the analysis was 1 year, for practical and computational reasons. The deviation due to this approximation was found to be negligible, because the error induced by the use of a yearly step was in the worst case < 3% compared with using 1 day as the time step.

### Statistical methods and models

Because longitudinal data were available for cadmium intake, assessed at three different occasions, the magnitude of the within-subject variation in dietary cadmium intake could be assessed by analysis of covariance (ANCOVA). More specifically, we performed a trend analysis to estimate any linear trend over time of both cadmium intake and cadmium intake per kilogram of body weight. A linear regression of intake was then fitted against age over the three time points, with a random effect on the subject and accounting for inter occasion variability with a fixed effect on the time point (using SAS proc MIXED; SAS version 9.1; SAS Institute Inc., Cary, NC, USA). Body weights were available at the three time points.

We chose an additive normal model to describe measurement error and residual variance. A Bayesian approach (Gelman et al. 2004; Wakefield et al. 1994) was used to perform the statistical inference, and posterior means were used for estimates. The Bayesian inference is an efficient alternative to the maximum likelihood approach, especially in the context of hierarchical (population) nonlinear models as presented here. Because it allows easy integration of prior information on parameters or constraints on them, identifiability (or separability) issues may be prevented. More details on Bayesian inference can be found in the references cited previously, and in Bernillon et al. (2000) regarding its specificity to TK modeling.

For the one-compartment model, the parameters to be estimated were *t*_1/2_ and *f_k_* × *f_u_* aside from the population parameters. In the Bayesian setup, prior distributions need to be defined on each model parameter. We set a noninformative (uniform) prior between 5 and 35 years for individual *t*_1/2_ values in order to constrain the estimation only within biologically plausible values. An informative prior was set over *f_k_* × *f_u_* in order to integrate the prior knowledge on the cadmium-related and physiologic parameters described above. This prior was set to a normal distribution (truncated at zero) centered on the central value given by the literature, and with CV = 30% covering the range of possible values for *f_k_*.

For the eight-compartment model, we fitted only the two most sensitive parameters (out of the 29) for computational reasons. They corresponded to the absorption fraction and the fraction transferred from plasma to extravascular fluid, and both were found to be slightly higher than the default values. For the Bayesian statistical analysis, we used only noninformative (flat) priors (restricted to positive values); that is, no information other than data was used to make the inference.

Monte Carlo Markov chains were used for the fitting using WinBUGS version 1.4 for the one-compartment model (Lunn et al. 2000), and an ad hoc Metropolis-Hastings algorithm (random walk) coded in Matlab release 14 (Mathworks, Inc., Natick, MA, USA) for the eight-compartment model. We used the Gelman-Rubin (Gelman et al. 2004) coefficient to assess the convergence of Markov chains. All other statistical exploratory analyses (tests, regressions and data management) were performed with SAS version 9.1. All graphs and simulations were made using Matlab.

The model selection procedure was defined as follows. We fitted both models as previously described, without any population variability. The mean predictions from the two fitted models were then compared. Only in the case of a substantially better prediction power would the eight-compartment model be the model of choice; otherwise, the one-compartment model would be used. Subsequently, we added variability parameters to the chosen TK model. We did not prespecify the intraindividual variability in urinary cadmium; instead, we performed a sensitivity analysis on the final model to assess the consequence of assuming such an additional interoccasion variability. A population layer was added to describe the interindividual variability (Rowland et al. 1985; Wakefield et al. 1994), by adding a lognormal random effect on half-life parameter(s).

In order to validate the final population model, two sensitivity analyses were performed: *a*) Observed urinary concentrations were plotted against the mean individual predicted ones, and *b*) A cross-validation was performed by first fitting on two-thirds of the data and then plotting the population predictions against the remaining one-third observed concentrations to detect any undesirable pattern or bias.

Last, the model parameters were set to their statistical estimates and Monte Carlo simulations were run in order to derive the population distribution of daily cadmium intake required to reach a given level of urinary cadmium after lifetime exposure.

## Results

### Cadmium intake variability

**Figure 1:**
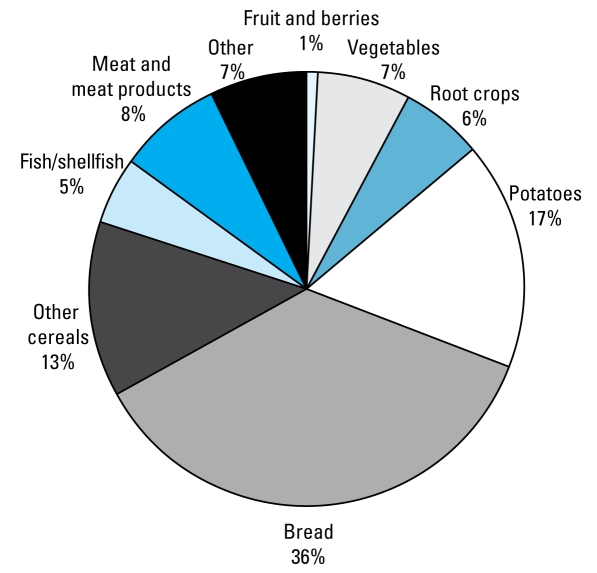
Major dietary sources of cadmium (%) in 680 never-smoking women at baseline (1987) of the SMC.

Table 1 presents the major characteristics of the 680 women included in the study. The overall average daily cadmium intake at the three different occasions was 14 μg, similar to that previously reported at baseline for all 30,210 postmenopausal women (Åkesson et al. 2008). Figure 1 presents the major dietary sources of cadmium intake at baseline. Because these results agreed with those of the entire population-based cohort (data not shown), we considered the 680 women in the present study representative of upper middle-age Swedish women.

We found the test for the time trend analysis (ANCOVA) to be significant for cadmium intake (*p* = 0.02) but not significant for the cadmium intake per kilogram of body weight (*p* = 0.17). This result supported our assumption of constant cadmium intake per kilogram of body weight, at least for the adult population. The between-occasion SD for the daily intake was found to be about 2 μg/day (i.e., about 15% variability between years) when assuming that this variability is constant over time. However, such a between-year variation is expected to underestimate the between-days intake variability. In addition, the FFQ used for the intake assessment may underestimate the intraindividual variation in food intake, because in most cases we predefined the response categories. As a consequence, a 25% variability of intraindividual daily intake was assumed (with normal distribution) and integrated into the one-compartment model.

### Model comparison

**Figure 2:**
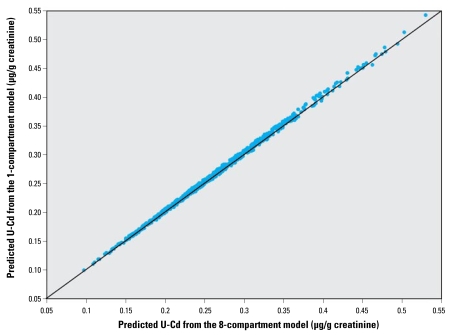
Comparison of individual urinary cadmium (U-Cd) predictions using the one-compartment model and the eight-compartment model.

The mean predictions of urinary cadmium using the two models were very close (Figure 2). This finding is consistent with a similar conclusion in WHO/IPCS (1992) regarding model comparison between a one-compartment model and a PBTK model (the Kjellström and Nordberg model in that case). Because the two models result in essentially the same predictions, the eight-compartment cannot be said to have a substantially better prediction power. Therefore, we chose the one-compartment model in the subsequent calculations.

### Model fitting based on the SMC data

**Figure 3:**
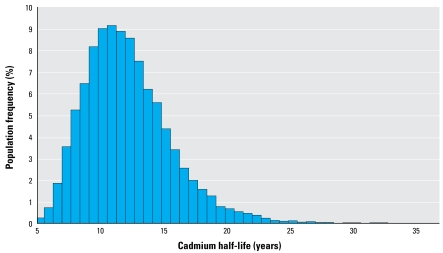
Estimated distribution of apparent half-life of kidney cadmium in the study population based on the one-compartment model and assuming lognormality.

The population one-compartment model was then fitted to the SMC data. We ran three parallel Markov chains (> 300,000 simulations) in order to obtain the posterior distributions of parameters and thereafter their estimates. Thinning was set to 10, and the 150,000 first simulations were viewed as burning time (Gelman et al. 2004; Lunn et al. 2000). Table 2 reports all statistical estimates. The population mean half-life of cadmium in the kidney was estimated to be about 11.6 years [95% confidence interval (CI) = 10.1–14.7]. Figure 3 shows the distribution of the half-life in the population (assumed to be lognormal, truncated to a range of 5–35 years).

**Figure 4:**
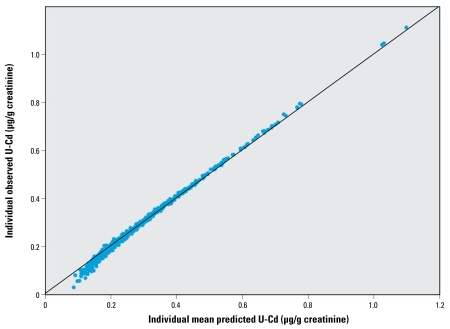
Measured individual urinary cadmium concentrations (U-Cd) versus predicted individual concentrations using the one-compartment model.

**Figure 5:**
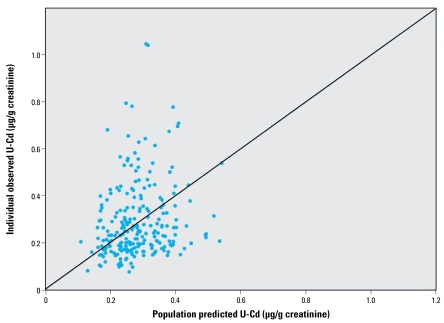
Cross-validation plot of observed individual urinary cadmium (U-Cd) concentrations versus population mean predictions around the population mean. The model was fitted on two-thirds of the data, and the population mean predicted was plotted against the remaining one-third observed.

The concordance between measured and predicted individual urinary cadmium concentrations was very high, as shown in Figure 4. The population-based model fitted well to the empirical data, except at the lowest concentrations (< 0.1 μg/g creatinine). In the cross-validation (Figure 5), the observed versus predicted population variability around the population mean shows no obvious bias or skewness.

### Derivation of the link between cadmium intake and lifetime exposure

**Figure 6:**
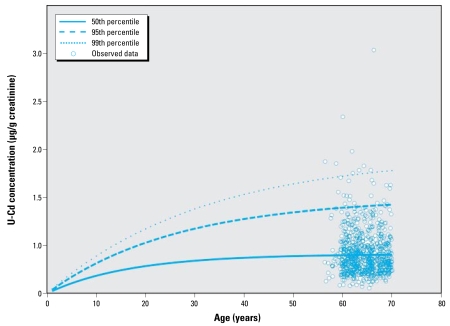
Predicted urinary cadmium concentrations corresponding to a daily cadmium intake of 0.3 μg/kg body weight over 70 years in the 50th, 95th and 99th percentiles of the population, overlaid by the data (circles)after adjustment to an intake of 0.3 μg/kg body weight.

Based on the estimated parameters, we ran Monte Carlo simulations in order to predict the population variation in urinary cadmium as a function of lifetime exposure, for a given daily intake. Figure 6 shows the predicted urinary cadmium concentrations corresponding to a daily cadmium intake of 0.3 μg/kg body weight over 70 years in the 50th, 95th, and 99th percentiles of the population. The upper percentiles represent the individuals at most risk for high urinary cadmium concentrations, (mainly) because of long retention time in the body.

**Figure 7:**
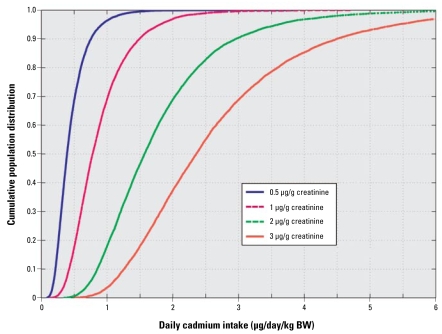
Cumulative population distribution of daily dietary cadmium intake corresponding to urinary cadmium concentrations of 0.5, 1, 2, and 3 μg/g creatinine), in a 50-year-old individual. The curves show, for each population percentile (*y*-axis), the maximum dietary cadmium intakes (*x*-axis) allowed in order not to exceed the predefined urinary cadmium concentrations.

Based on the model, the population distribution in the daily cadmium intake corresponding to a given level of urinary cadmium could also be obtained. Thus, we calculated the population variation in dietary cadmium intake corresponding to urinary cadmium concentrations of 0.5, 1, 2, and 3 μg/g creatinine, in a 50-year-old individual with 70 kg body weight (Figure 7), to be used for risk assessors. The age of 50 years was chosen because urinary cadmium is considered at its maximum at this age in previous risk assessments of cadmium (WHO/FAO 2004). The curves show, for each population percentile, the maximum dietary cadmium intakes allowed in order not to exceed the predefined urinary cadmium concentrations. Thus, in order to remain below, for example, 1 μg cadmium/g creatinine in urine in 50% of the population by the age of 50 (average urinary cadmium is 1 μg/g), the average daily dietary cadmium intake should not exceed about 0.8 μg cadmium/kg body weight. We found the corresponding intake for the 95th percentile of the population remaining below 1 μg/g to be about 0.4 μg cadmium per kilogram of body weight and per day.

### Sensitivity analyses

Unlike the eight-compartment model, the one-compartment model allowed all parameters to be statistically estimated, implying that the results must not depend on fixed default values. Therefore, no sensitivity analysis was required in that respect. However, we performed sensitivity analyses with respect to the modeling assumptions, namely, the *a priori* information chosen for the Bayesian analysis, the choice for dietary cadmium intake variability, and the description of intraindividual variability.

The Bayesian approach used allowed incorporation of some *a priori* information taken from the literature but still considering the uncertainty (CV = 30%, normal distribution). We performed a sensitivity analysis with respect to the choice of such information, using a much less informative (prior) distribution on the *f_k_* parameter (using CV = 100%). The parameter estimates were close to the original ones (< 5% difference), resulting in no major change in the model predictions. Similarly, the model fit was also performed without the constraint that the population mean half-life should be between 10 and 30 years. The resulting parameter estimates were also very close to the original ones. Table 3 summarizes the parameter estimates using those alternative prior distributions.

The interday variability in dietary cadmium intake could not be directly estimated from the data. To evaluate the impact of the assumption we used of 25% variability, a sensitivity analysis was performed. We set a lower bound for the variability to 15%, as actually estimated for the variation data over the three data collection periods for the dietary intakes. An upper bound of 50% as estimated in the NHANES III (1988–1994) study by Choudhury et al. (2001) was chosen, where the cadmium intake variability (interindividual intraindividual variability over the survey time) was about 50%. The results based on the assumed intake variability between 15% and 50% showed < 5% difference in all parameters estimated; that is, we found the model to be very robust.

**Figure 8:**
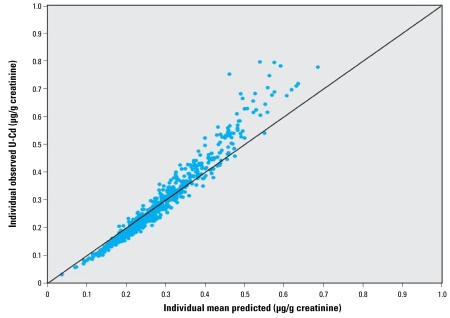
Measured individual urinary cadmium (U-Cd) concentrations versus individual predictions from the one-compartment population model, using additional 25% intraindividual variability on U-Cd.

Finally, a sensitivity analysis was also performed with respect to additional intra-individual variability of urinary cadmium concentrations, which could not directly be estimated from the data. In the final model, we estimated the residual variance to be about CV = 13% on average of the urinary cadmium measured. This variance encompasses mainly the intraindividual variability (e.g., intraoccasion variation), but also some measurement error. This may be too small, so some of this intraindividual variability may have been transferred into the population variability, hence inflating the variability shown in Figure 6. In order to assess what would be the outcome of a similar analysis but fixing some predetermined interoccasion variability, we added an additional variance term in the error model, which we set as a multiplicative (lognormal) error with CV = 25%, hence describing an additional 25% variability of urinary cadmium concentrations within subjects. A similar statistical inference was subsequently performed. The resulting fit was not as good as the fit without this new assumption, especially for urinary cadmium concentrations > 0.4 μg/g creatinine, leading to a consistent underestimation of the model (Figure 8). Although the data do not fully support this new assumption, the parameter estimates could be derived. We estimated the new mean half-life to be 11.6 years (95% CI = 9.3–16) and the new intersubject variability to 2.7 years (95% CI = 2–4). As expected, the intersubject variability had been transferred to intrasubject variability, although in a moderate way. The 5th percentiles of daily cadmium intake needed to achieve a given urinary cadmium value were seen to be relatively robust with respect to this extravariability assumption (~ 10% higher when such a variability is taken into account).

## Discussion

This study is the first to evaluate, on a population level, the variability in cadmium kinetics using paired individual data on dietary intake and urinary excretion. Our results clearly demonstrate the need to consider the large variation in cadmium kinetics when translating biomonitoring data such as urinary cadmium into data on dietary intake of cadmium. The one-compartment TK model proved to be as suitable as the eight-compartment model to fit urinary cadmium data and provides additional flexibility to allow for population variability modeling.

In this study of 680 Swedish women, the estimated average daily cadmium intake of 14 μg was similar to that observed in Europe and the United States in areas with no industrial cadmium contamination (Berglund et al. 1994; Egan et al. 2007; Larsen et al. 2002; Llobet et al. 2003; MacIntosh et al. 1996; Olsson et al. 2002; Thomas et al. 1999; Ysart et al. 2000). Furthermore, the dietary cadmium intake of the women was considered to be representative of upper middle-aged Swedish women (Åkesson et al. 2008).

The major advantage of the present study is the availability of both long-term intake and biomarker data at the individual level, allowing individual linking of intake to urinary cadmium concentrations and estimation of the population variations. This allowed, for the first time, the assessment of the between-person variability in cadmium TK at the population level, hence allowing the derivation of a robust model to be used in risk assessment. Figures 3 and 4 illustrate how the model can capture such variability. This procedure also permits the quantification of the link between cadmium intake and urinary cadmium at the individual level, making possible more exact individual predictions.

In a previous U.S. study (Choudhury et al. 2001; Diamond et al. 2003), the association between urinary cadmium and cadmium intake was assessed based on NHANES III (1988–1994). The study provided empirical mean estimates of urinary cadmium and cadmium intake over the U.S. population, stratified by age groups and by sex. Choudhury et al. (2001) and Diamond et al. (2003) also predicted the central tendency of urinary cadmium from the mean daily cadmium intake using the eight-compartment model as described by Nordberg et al. (1985). The main advantage of the U.S. study was that they were able to evaluate the entire U.S. population in terms of different age strata in both men and women and could assess and account for cadmium intake variability. On the other hand, although available, individual cadmium intake and urinary cadmium could not be paired for the evaluation of interindividual variability in toxicokinetics. The 24-hr recall method used for intake measurement in the NHANES survey may be a more imprecise proxy of long-term cadmium intake than the FFQ method used in the SMC study.

The urinary cadmium concentrations in the NHANES III data for women 40–60 years of age was about 0.55 μg/g creatinine, corresponding to a daily intake of 0.3 μg/kg body weight. By using the default parameter values in the eight-compartment model (with a 5% absorption coefficient), Choudhury et al. (2001) arrived at a predicted urinary cadmium concentration of 0.3 μg/g creatinine, which was in good agreement with the NHANES III measured average value for men but clearly underestimated the urinary concentrations for women. A likely explanation is the higher absorption rate in women (up to 10%). At the same intake level of 0.3 μg cadmium per day per kilogram of body weight, the predicted urinary cadmium based on our model was 0.39 μg/g creatinine for a median individual (50th percentile in Figure 6) but was 0.43 μg/g creatinine when we averaged the predictions over the population, which is consistent with the U.S. data. Thus, the results might indicate an average absorption rate in women > 5% but < 10%. The difference, although not statistically significant, between the 0.55 μg cadmium/g creatinine measured in urine in the NHANES III and the mean of 0.43 μg/g creatinine estimated by the model based on our data could also be due to other differences in population characteristics or differences in intake measurement methods.

Further strengths of our study were the large number of individuals (680) and our ability to create a detailed food-cadmium database based on the cadmium content in practically all foods available on the Swedish market. The data were provided by the NFA, which has monitored cadmium content in food (up to several hundred analyses per food item) repeatedly over the past decades (Becker and Kumpulainen 1991; Jorhem and Sundstrom 1993; Jorhem et al. 2001).

A possible limitation associated with the present study may be that the dietary cadmium intake was estimated based on an FFQ, providing a rather rough measure of the cadmium intake levels of the individuals. On the other hand, the validation based on the consumption of major cadmium-contributing foods in a subgroup of the women and that based on biological markers in another population were satisfactory. The use of the average cadmium concentration for each food item in the questionnaire was substantiated by the fact that there is no known industrial cadmium contamination of agricultural soil in the study area and no known geographical variation in cadmium content of consumed foods across Sweden. In addition, most foods are distributed throughout the country by wholesale companies, giving a fair representation of what the women in our study would encounter. We might, however, not have full access to all interday variability in the cadmium intake as assessed by the FFQ; instead, we estimated and accounted for the population variability in the half-life of cadmium in the kidney, in addition to the intraindividual intake variability that we fixed at 25%.

Furthermore, the assumption of constant cadmium intake per kilogram of body weight over a lifetime could also be challenged, especially for younger ages. However, considering the estimated half-life (~ 12 years) and the age range of our study population (> 50 years of age), even large variations of intake before 20 years of age are expected to have limited impact on the cadmium burden at older ages. Based on the model estimates, thus, a variation of 50% in cadmium intake until 15 years of age would, based on our model estimates, result in < 3% difference in the cadmium burden at 50 years of age. Finally, the adjustment to a constant energy intake of 1,700 kcal (mean intake recorded in the FFQ) may underestimate the cadmium intake, because energy expenditure can be expected to be up to 15% higher [WHO/FAO/United Nations University 2001].

Another limitation is that the population variability estimates may be biased because they derive from older women only. However, including women is appropriate for risk assessment purposes because women generally have higher concentrations of cadmium in blood, urine, and kidney than do men, because of higher gastrointestinal cadmium absorption (Vahter et al. 2007). Thus, women are considered more at risk of adverse effects of cadmium, and protecting them would likely protect the entire population.

Finally, other sources of variability and biases related to both TK and intake lead to variations over a lifetime that were not possible to account for.

Interestingly, because of the simple form of the model, any systematic bias made over the population on, for example, intake or absorption rate will not have any impact on the final outputs because it would only correspond to an additional factor within *f_k_* × *f_u_* to be globally estimated. As a consequence, the biological interpretation of the *f_k_* × *f_u_* results may not be meaningful. This illustrates a common difference between such a population one-compartment model and more physiologically based models in which each parameter clearly corresponds to one physiologic variable. Similarly, all the population variability has been imputed to the half-life parameter. Accounting for an additional population variability, for example, on absorption (i.e., on *f_k_* × *f_u_*) leads to an overparameterized model that cannot be fitted to the available data. This choice of parameterization may result in some inflation of the population variance of the cadmium half-life, which is of no consequence for the risk assessment objective but should be acknowledged for the biological interpretation. Indeed, this apparent variability may still encompass some variability, for example, in absorption rate. Figures 4 and 6 indicate that most population variability has been captured by the model.

## Conclusions

The population modeling of a large database of individually matched long-term cadmium intake and urinary cadmium concentrations was a helpful tool for human risk assessment. More specifically, we showed the following:

- A one-compartment TK model could provide prediction of individual urinary cadmium sensibly similar to that of a PBTK model.
- A population one-compartment model allowed integration and quantification of the between-person variability in cadmium TK, in particular, the population mean half-life of cadmium in the kidney.
- The population distribution of daily cadmium intakes corresponding to a predefined urinary cadmium concentration could be derived in a robust manner. The derivation of a maximum daily cadmium intake would ensure that 95% of the most vulnerable population subgroups do not reach a predefined level of cadmium body burden, which is crucial to support risk assessment.

## Figures and Tables

**Table 1:** Main characteristics of the 680 women participating in the study.

Characteristic	Mean	Median	Range
Age (years)	64	64	56–70
Weight (kg)	69	68	36–124
Daily dietary cadmium intake^a^			
1987–2007 (μg)	14	14	9–21
1987–2007 (μg/kg body weight)	0.2	0.2	0.1–0.4
1987 (μg)	15	15	8–26
1997 (μg)	13	13	3–24
2004–2007 (μg)	13	13	7–27
Urinary cadmium (μg/g creatinine)	0.34	0.31	0.09–1.23

a Adjusted for total energy intake of 1,700 kcal using the residual regression method.

**Table 2:** Statistical estimates of model parameters and their 95% CIs.

Parameter	Mean posterior estimate	95% CI
Population mean *t*_1/2_ (years)	11.6	10.1–14.7
Population SD *t*_1/2_ (years)	3.0	2.5–4.0
Factor (*f_k_* × *f_u_*)	0.005	0.0031–0.0063
Residual variance (μg/g creatinine)^2^	0.0012	0.0008–0.0020

**Table 3:** Comparison of the parameter estimates (posterior means and 95% CIs) when using the original prior, when using the same prior but with inflated variance (CV = 100% instead of 30%), and when using a prior on the population mean *t*_1/2_ without any bounds.

	Mean posterior (95% CI)		
Estimate	Using the original priors	Prior on (*f_k_* × *f_u_*) with inflated variance (CV = 100% instead of 30%)	Prior on population mean *t*_1/2_ not bounded by 10 and 30 years
Population mean *t*_1/2_ (years)	11.6 (10.1–14.7)	11.5 (10.1–16.3)	11.3 (9.5–15.3)
Population SD *t*_1/2_ (years)	3.0 (2.5–4.0)	3.0 (2.5–4.5)	2.9 (2.3–4.2)
Factor (*f_k_* × *f_u_*)	0.005 (0.0031–0.0063)	0.005 (0.0026–0.0063)	0.0052 (0.0029–0.0070)
Residual variance (μg/g creatinine)^2^	0.0012 (0.0008–0.0020)	0.0012 (0.0008–0.0020)	0.0012 (0.0008–0.0013)
